# Gastric Neuroendocrine Tumor

**DOI:** 10.1055/s-0041-1731427

**Published:** 2021-07-19

**Authors:** Naresh Kargwal, Viraj Panda, Abhijeet Jha, Chandra Bhushan Singh

**Affiliations:** 1 Department of General Surgery, Maulana Azad Medical College, New Delhi, India

**Keywords:** carcinoid, neuroendocrine, tumor, gastric

## Abstract

Gastric neuroendocrine tumor (gNET) is a rare carcinoid of the stomach whose incidence is increasing due to widespread use of upper gastrointestinal endoscopy (UGIE). There are four types of gNETs with different management strategies and prognosis. Here, we present a patient who came with abdomen pain and intermittent melena. UGIE showed a sessile polyp in the stomach. The patient subsequently underwent polypectomy and was symptomatically relieved.

Gastric neuroendocrine tumors (gNETs) were first reported by Askanazy in 1923. 1 gNETs have an incidence of 1 to 2 cases per 1,000,000 people 2 as per a study by Dias et al. It accounts for 8.7% of all neuroendocrine tumors (NETs) 3 and 1.8% of all gastric tumors. 2 They are rare and have an indolent behavior with a neuroendocrine differentiation. Their incidence is increasing due to the widespread use of upper gastrointestinal endoscopy (UGIE) and the technical refinement of endoscopists. 2 Therefore, a suspicion of a NET must be kept in mind for a patient presenting with self-limiting hematemesis with a polyp on UGIE.

## Case Presentation

### History and Examination

A 38-year-old man presented to the surgery clinic with complaints of upper abdomen pain for 1 year. The pain was dull aching with episodes of increased severity lasting for 1 to 2 hours. The pain was relieved with oral analgesics. This was associated with occasional black tarry stools. The patient also complained of easy fatigability, breathlessness, and palpitations. He had a history of repeated blood transfusions for severe anemia. He was a chronic smoker and tobacco chewer.

His clinical examination revealed pallor. The rest of the examination was unremarkable.

### Investigations

The patient had severe anemia with hemoglobin of 5.7 g/dL for which he was transfused with two units of packed cells. Subsequently, his hemoglobin improved to 8.3 g/dL.

UGIE showed a 1 × 1 cm polyp with central umbilication along the greater curvature of the body of the stomach. There was no active bleeding. The mucosa of the rest of the stomach appeared normal. An impression of a gastrointestinal stromal tumor or NET was kept. The serum chromogranin A (CgA) level was 304 ng/L (normal value = 28–94 ng/mL).

**Fig. 1:**
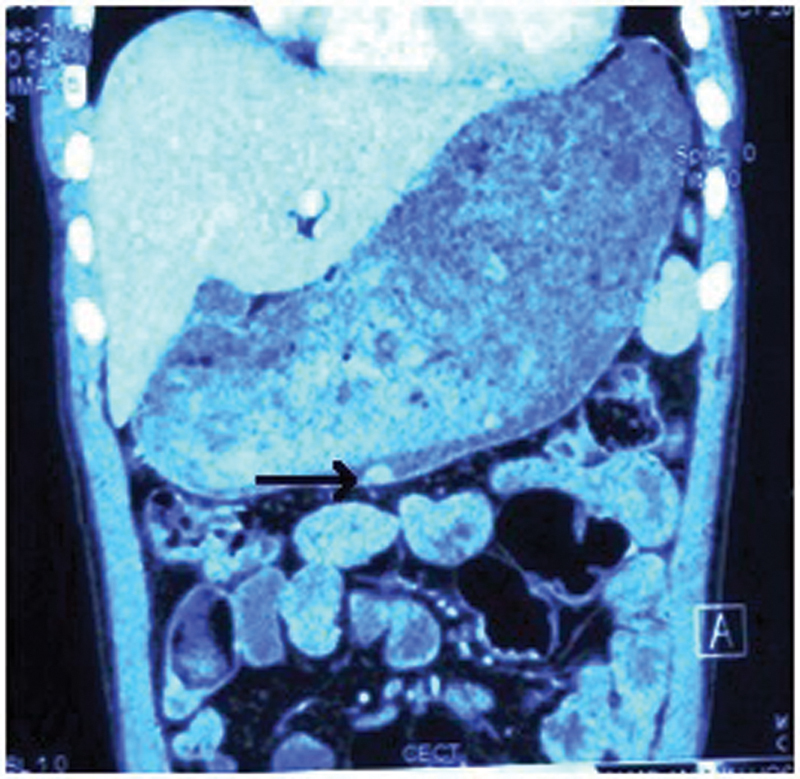
Contrast enhanced computed tomography of the abdomen showing polypoidal lesion on the greater curvature of the stomach (black arrow).

**Fig. 2:**
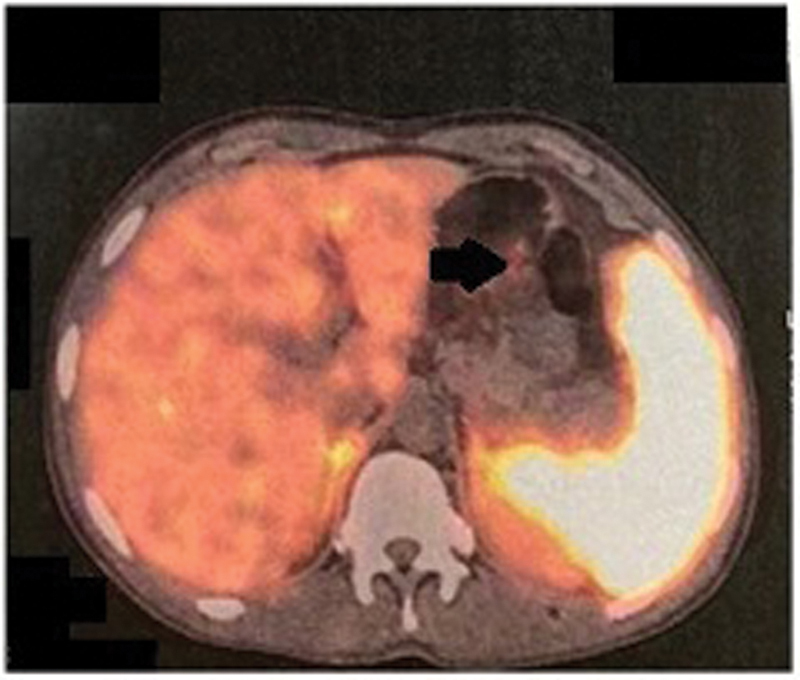
Gd-68 DOTATATE scan showing somatostatin receptor expressing polypoidal lesion on the greater curvature of the stomach (black arrow).

Contrast enhanced computed tomography (CECT) showed evidence of a hyperenhancing polypoidal lesion along the greater curvature measuring 1.1 × 0.7 cm ( Fig. 1 ). There were no enlarged lymph nodes visualized. Gd-68 DOTATATE scan showed a somatostatin receptor (SSTR) expressing small polypoidal soft tissue density in the body of the stomach along the greater curvature ( Fig. 2 ).

**Fig. 3:**
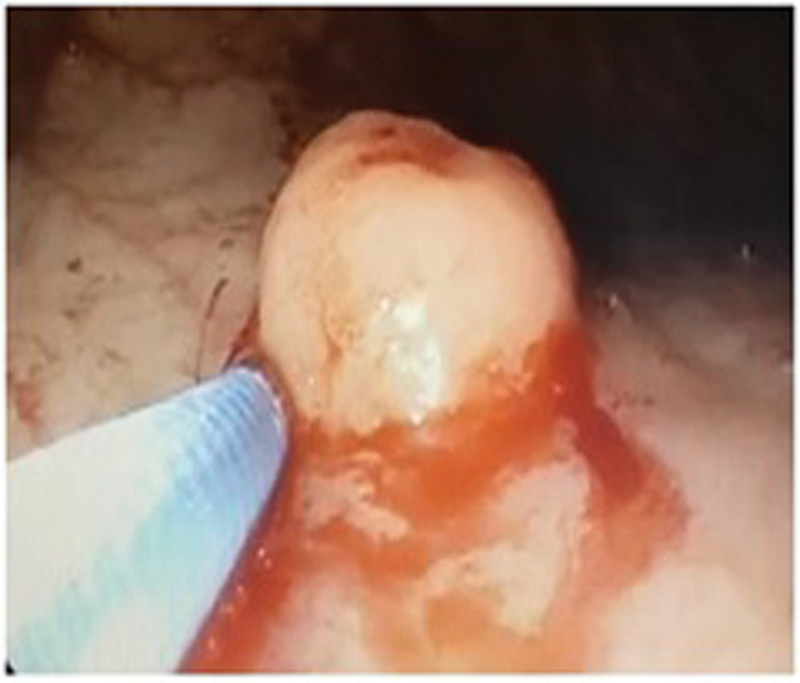
Upper gastrointestinal endoscopy showing polyp.

Subsequently, the patient underwent resection of the polyp under a second endoscopy ( Fig. 3 ) which was sent for histopathological examination (HPE).

**Fig. 4:**
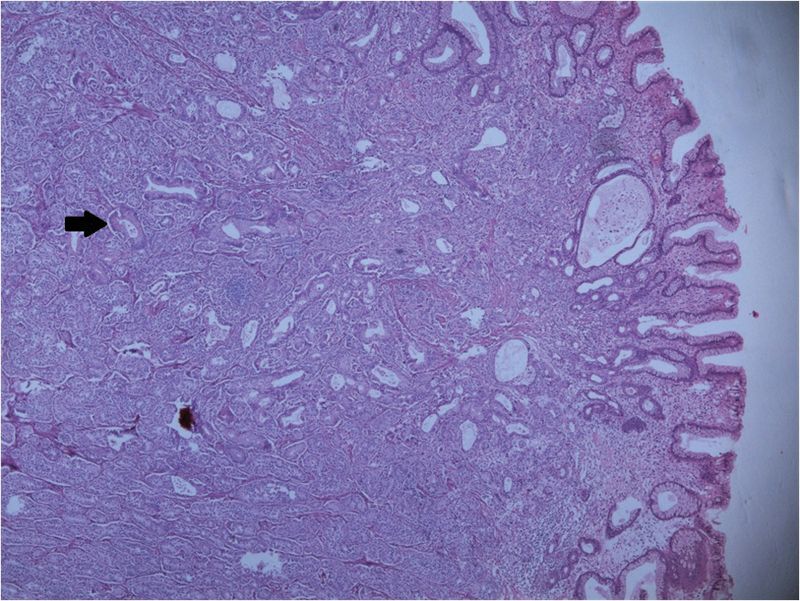
Histopathological examination showing tumor cells arranged in acinar pattern (black arrow).

**Fig. 5:**
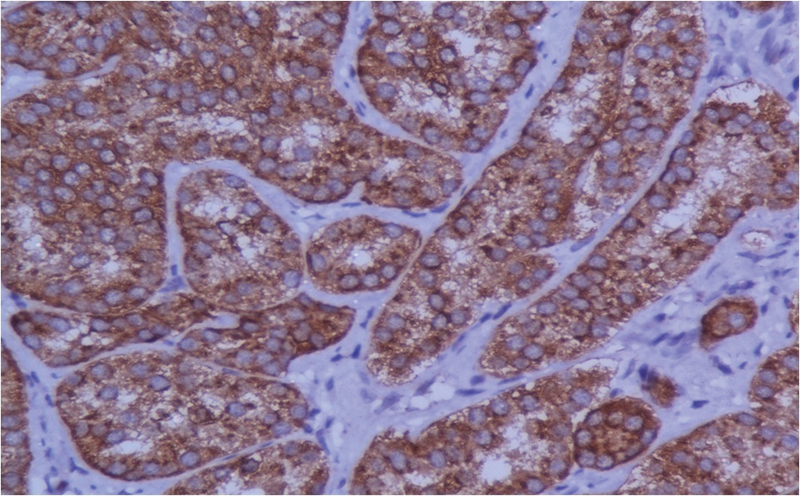
Chromogranin positive tumor cells.

HPE showed a polypoidal structure lined by gastric mucosa ( Fig. 4 ). The mucosa and submucosa were infiltrated by a tumor that was arranged in an acinar pattern. The tumor cells were monomorphic with a moderate amount of cytoplasm and salt and pepper nuclear chromatin. Occasional mitotic figures were seen. The base of the polyp was free of tumor cells. The tumor was diffusely positive for synaptophysin and CgA on immunohistochemistry ( Fig. 5 ). Ki-67 index was 3%. The impression made from the above findings was NET of the World Health Organization (WHO) grade II.

The patient was immediately relieved of symptoms following polypectomy and was discharged 2 days later. His hemoglobin after 2 weeks on follow-up was 10.5 g/dL. He is currently asymptomatic.

## Discussion

The majority of gNETs are neoplasms derived from the enterochromaffin-like cells (ECL cells) of the gastric mucosa. The cells comprising these tumors are avid for salts of silver. These ECL cells play an important role in regulating HCl secretion in the stomach. 4 An extremely rare form of gNET (type IV) may also arise from the gastrin or serotonin producing endocrine cells of the stomach. 5

Patients with this pathology present with upper abdomen pain, bloating, nausea or vomiting, symptoms of anemia, weight loss, or hematemesis. Further evaluation may reveal an associated condition such as Zollinger–Ellison's syndrome or multiple endocrine neoplasia type 1. 4

Gastric polyps may incidentally be found on UGIE. Their prevalence on UGIE is approximately 6%. The most common type of polyp of stomach is fundic polyp comprising 70 to 90% of all polyps. They occur due to long-term proton-pump inhibitor (PPI) use. Polypectomy is recommended for polyps more than 1 cm in size or with a suspicion of malignancy. Mucosal sampling is also done along with polypectomy to rule out atrophic gastritis and *Helicobacter pylori* infection. 6

gNETs are classified into four distinct types with different characteristics, treatment, and prognosis ( Table 1 ).

**Table 1:** Types of gNETs 7

Features	Type 1	Type 2	Type 3	Type 4
Prevalence among gNETs	70–80%	5–10%	15–20%	Rare
Number of lesions	Multiple	Multiple	Single	Single
Size	1–2 cm	1–2 cm	>2 cm	Largest 16 cm
Other symptoms	Autoimmune polyglandular syndrome	MEN-1 (gastrinoma)	–	–
Serum gastrin level	Raised	Raised	Normal	Normal
Gastric pH	Raised	Reduced	Normal	Normal
Underlying mucosa	Atrophic	Hypertrophic	Normal	Normal
Invasion	Mucosa/submucosa	Mucosa/submucosa	Any depth	Any depth
Metastasis				
Lymph nodes	5–10%	10–20%	50–100%	–
Liver	2–5%	10%	22–75%	100%
Prognosis	Excellent	Very good	Poor	Poor
Proliferation index (Ki-67)	<2%	<2%	>2%	>30%
Immunohistochemistry	CgA, NSE, VMAT 2 positive	CgA positive	CgA negative	Synaptophysin, NSE, PGP 9.5 positive CgA negative

Abbreviations: CgA, chromogranin A; gNET, gastric neuroendocrine tumor; HPF, high-power field; MEN-1, multiple endocrine neoplasia type 1; NSE, neuron-specific enolase; PGP, protein gene product; VMAT, vesicular monoamine transporter.

### Immunohistochemistry

Confirmation of NETs is brought about by immunohistochemical analysis with the help of CgA and synaptophysin. This also helps in classifying lesions according to the WHO histological grades. The proliferative index Ki-67 and the number of mitoses per high magnification field help predict the prognosis. Prognosis and risk of metastasis may also relate to p53 and serum enolase biomarkers. The positive predictive rates for CgA is 40%, 60% for synaptophysin, 60% for CD56, 40% for neuron-specific enolase, and 100% for p53. 8

### Staging

CECT of the abdomen is necessary for types I and II gNETs more than 2 cm and for all type III lesions. 2 NETs are rich in SSTRs 2 and 5 which are detected by somatostatin scintigraphy. Octreoscan is a technetium-based somatostatin scintigraphy test with a high sensitivity and specificity. 9 A recently developed gallium-labeled octreotide captured by positron emission tomography scans shows superiority over the octreotide scintigraphy. 2

### Treatment

The treatment of gNETs depends on the type, extent, grade of differentiation, and presence of poor prognostic factors. The WHO classified these neoplasms into three histological degrees with graded prognosis ( Table 2 ).

**Table 2:** WHO 2010 classification of NETs 5

Differentiation	Grade 1 (well)	Grade 2 (moderately)	Grade 3 (poorly) a
Metastases	−	−	+
Muscularis propria invasion	−	±	+
Tumor size (cm)	≤2	>2	Any
Mitoses/10 HPF b	<2	2–20	>20
Ki-67 index %	≤2	3–20	>20
Angioinvasion	Never	Late	Always

Abbreviations: HPF, high-power field; NET, neuroendocrine tumor; WHO, World Health Organization.

a Grade 3 is divided into small cell and large cell neoplasms.

b HPF: High Power Field.

Poor prognostic factors include: lesion ≥2 cm, deep submucosal invasion or beyond, Ki-67 ≥3%, lymphovascular invasion, poorly differentiated and presence of atypia or necrosis. 2

Carcinoid crisis is provoked by tumor manipulation before or during surgery that can be prevented by subcutaneous or intravenous octreotide. 2

The first-line treatment for any polyp that is more than 1 cm or suspicious of malignancy is endoscopic polypectomy. Endoscopic polypectomy is adequate for types 1 and 2 gNETs. Further surgical treatment is necessary for type 3 gNETs and type 1 or 2 gNETs where endoscopic treatment is not feasible or there is presence of poor prognostic factors. Total or subtotal gastrectomy allows adequate removal of G-cells that is not attainable by antrectomy. Lymphadenectomy is added in the presence of poor prognostic factors or extragastric disease. 2 Octreotide decreases the gastrinemia but discontinuation of this treatment allows a rise in serum gastrin levels at 1 year follow-up. This treatment is reserved for patients unfit for surgery. 10 Vitamin B12 supplementation is given in all cases. 2

The latest development includes netazepide (YF476) that is a potent and highly selective cholecystokinin 2 receptor antagonist belonging to the benzodiazepine class. They cause regression of type 1 gNETs and normalize CgA levels. 6

Extrahepatic metastasis or recurrent symptomatic disease is treated with systemic therapy consisting of cytotoxic chemotherapy (streptozocin combined with 5-fluorouracil or cyclophosphamide, doxorubicin mono drug or with 5-fluorouracil, dacarbazine or temozolomide, oxaliplatin with capecitabine or 5-fluorouracil with leucovorin) or molecular-targeted agents (bevacizumab, sorafenib, sunitinib, pazopanib, and everolimus). 11 These tumors also reportedly benefited from targeted radiotherapy with 177Lu octreotide. 12

#### Carcinoid Syndrome

Carcinoid syndrome occurs in type 3 gNETs due to the release of histamine from the tumor cells. This causes erythema, itching, diarrhea, and bronchospasm. Symptoms are controlled with somatostatin analogs (octreotide or lanreotide) and low-dose interferon-alfa for refractory cases. 3

### Follow-up

Median survival of gNETs ranges from 13 months to more than 10 years. 2

The recommendation of the National Comprehensive Cancer Network for follow-up consists of history, physical examination, UGIE, abdominal CECT or magnetic resonance imaging, and serum CgA every 6 months for 1 to 2 years, annually for 4 years and then every 2 years until 10 years after surgery. Types I and II lesions <2 cm and without poor prognostic factors or features suspicious of malignancy may only be followed with history, physical examination, and UGIE every 6 to 12 months. 2 Serum CgA is used to monitor any recurrence following chemotherapy. However, somatostatin analogs and PPIs may alter these levels thereby reducing their sensitivity.

**Fig. 6:**
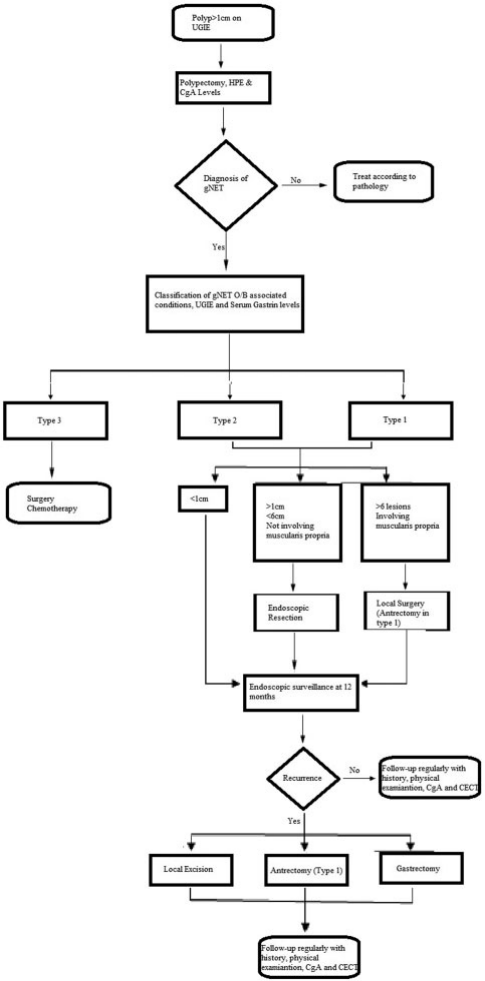
Treatment algorithm for gNETs. CgA, chromogranin A; gNET, gastric neuroendocrine tumor; HPE, histopathological examination; UGIE, upper gastrointestinal surgery.

Overall, the following algorithm may be used to treat gNETs ( Fig. 6 ).

## Conclusion

gNETs consist of a rare complex disease that includes different subtypes with distinct management and prognosis. Correct identification of the clinical type and histological grade allows for a tailored management. A suspicion of gNET must be kept in mind in a patient presenting with vague symptoms of abdomen pain and self-limiting hematemesis with a polyp on UGIE.
